# Supplementary material for: Constructing personalized characterizations of structural brain aberrations in patients with dementia using explainable artificial intelligence
Source: NPJ Digit Med. 2024 May 2;7:110. doi: 10.1038/s41746-024-01123-7 (PMC11066104; doi:10.1038/s41746-024-01123-7)
Supplement: Supplementary file 1 — Supplementary Figures and Tables [file 41746_2024_1123_MOESM1_ESM.pdf]

Supplementary Materials for

Constructing personalized characterizations of structural brain aberrations in dementia patients with explainable artificial intelligence

Esten H. Leonardsen et al.

Corresponding author: estenhl@uio.no

Contents

List of supplementary figures

1 Test performance for the dementia classifiers . . . 3
2 Example relevance maps . . . 4
3 Average relevance maps . . . 5
4 Statistical map from GingerALE . . . 6
5 Distributions of dementia predictions in MCI subsets . . . 7
6 Visualization of PCA components . . . 8
7 Correlations between PCA components . . . 9
8 Neuropsychological tests and PCA components . . . 10
9 Results from the hyperparameter search . . . 11
10 Region-wise comparison of $\bar{R}_{dementia}$ and G . . . 12

List of supplementary tables

1 Overview of datasets . . . 13
2 Inclusion criteria for participants . . . 14
3 Predictive performance of dementia classifiers by fold . . . 15
4 Effects of various covariates on the dementia prediction $\hat{y}$ . . . 16
5 Overview of survival analyses . . . 17
6 Predictive performance of progression models . . . 18
7 Neuropsychological assessments . . . 19
8 Neuropsychological test scores . . . 20
9 Dementia classifier hyperparameters . . . 21

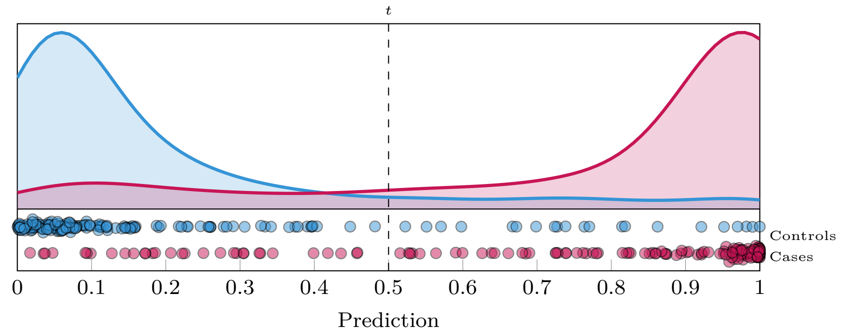

(a) Predictions

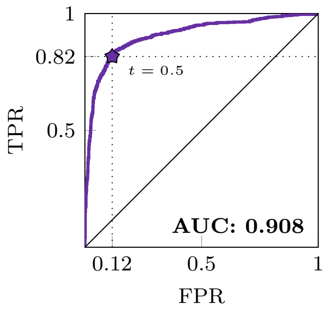

(b) ROC Curve

Predicted / Observed

	**0**	**1**
**0**	754	100
**1**	157	697

Accuracy: 84.95%

(c) Confusion matrix

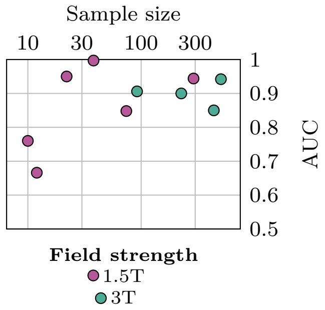

(d) AUC per site

Supplementary Figure 1: Predictive performance of the best performing dementia classifiers, combined across each out-of-sample test fold.

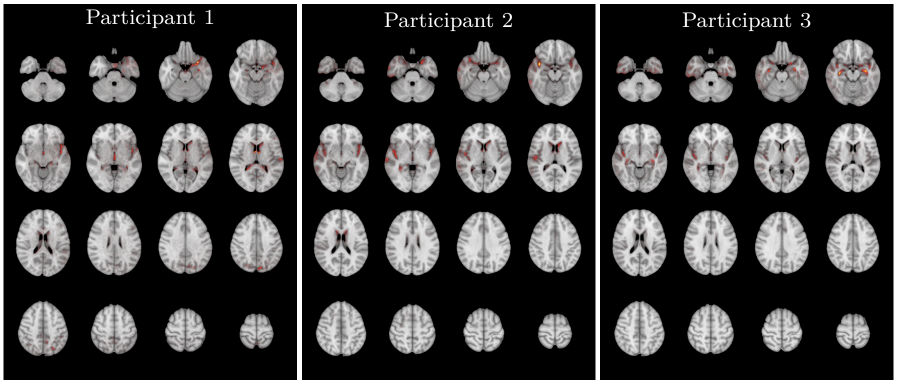

Supplementary Figure 2: Example relevance maps produced by the pipeline for three randomly selected dementia patients.

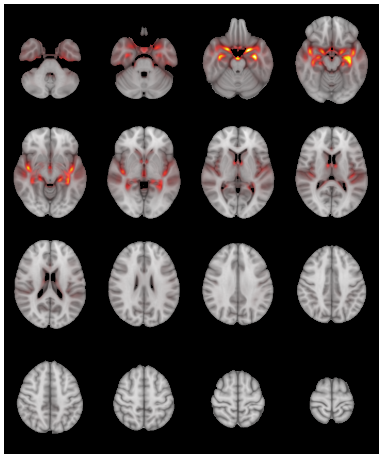

(a) Dementia

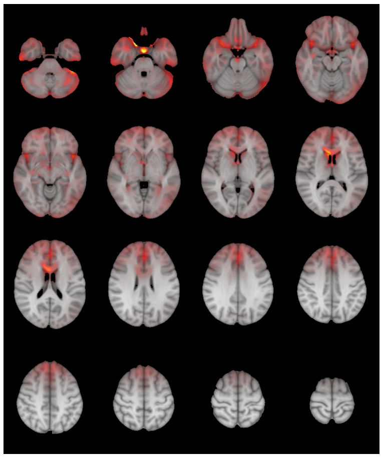

(b) Sex

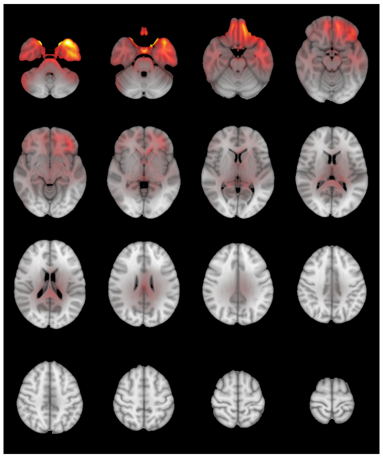

(c) Random weights

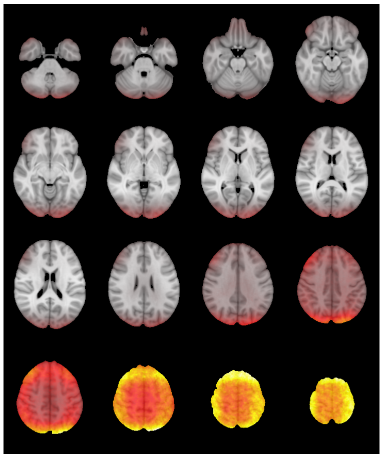

(d) Randomized images

Supplementary Figure 3: Average voxel-wise relevance maps produced for the four pipelines across all correctly predicted dementia patients.

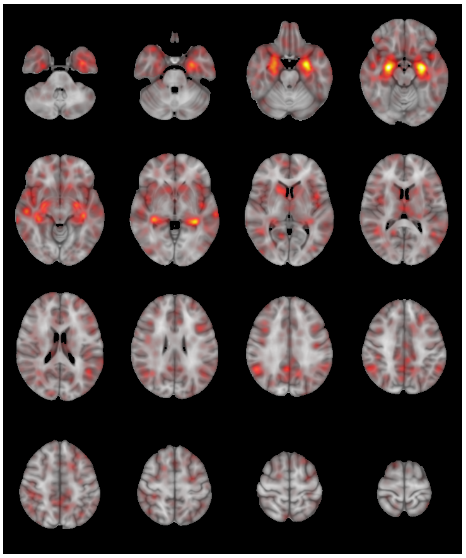

Supplementary Figure 4: Statistical map of dementia-related pathology generated by a GingerALE meta-analysis of 124 articles.

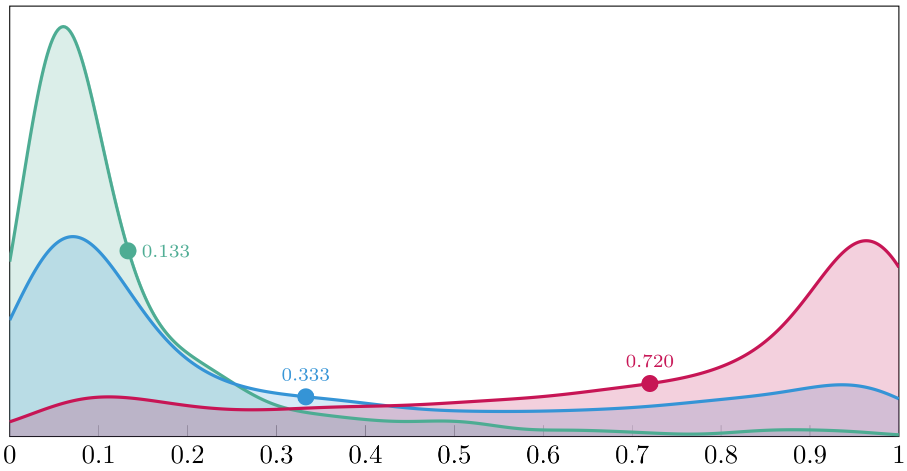

(a) All timepoints.

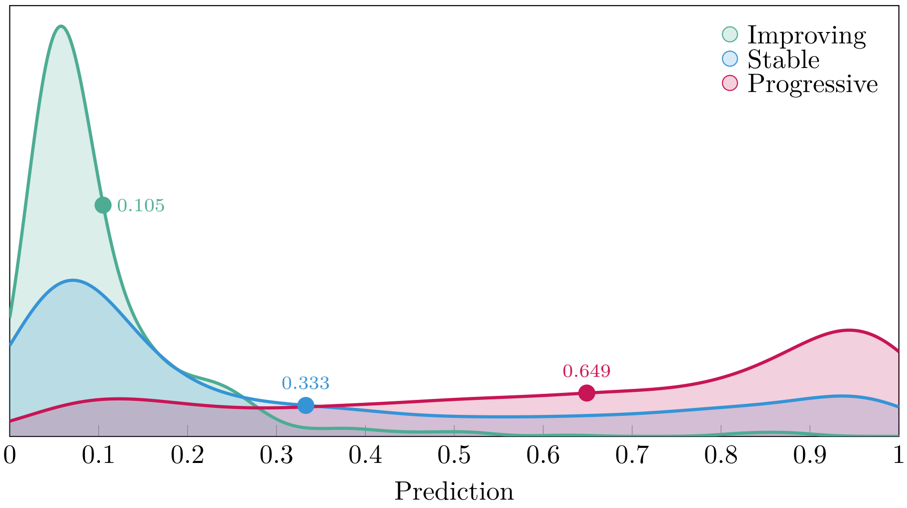

(b) Only timepoints when the patient got an MCI diagnosis.

Supplementary Figure 5: Distribution of predictions from the dementia classifier in the three MCI subsets. The marks indicate the mean prediction in each subset.

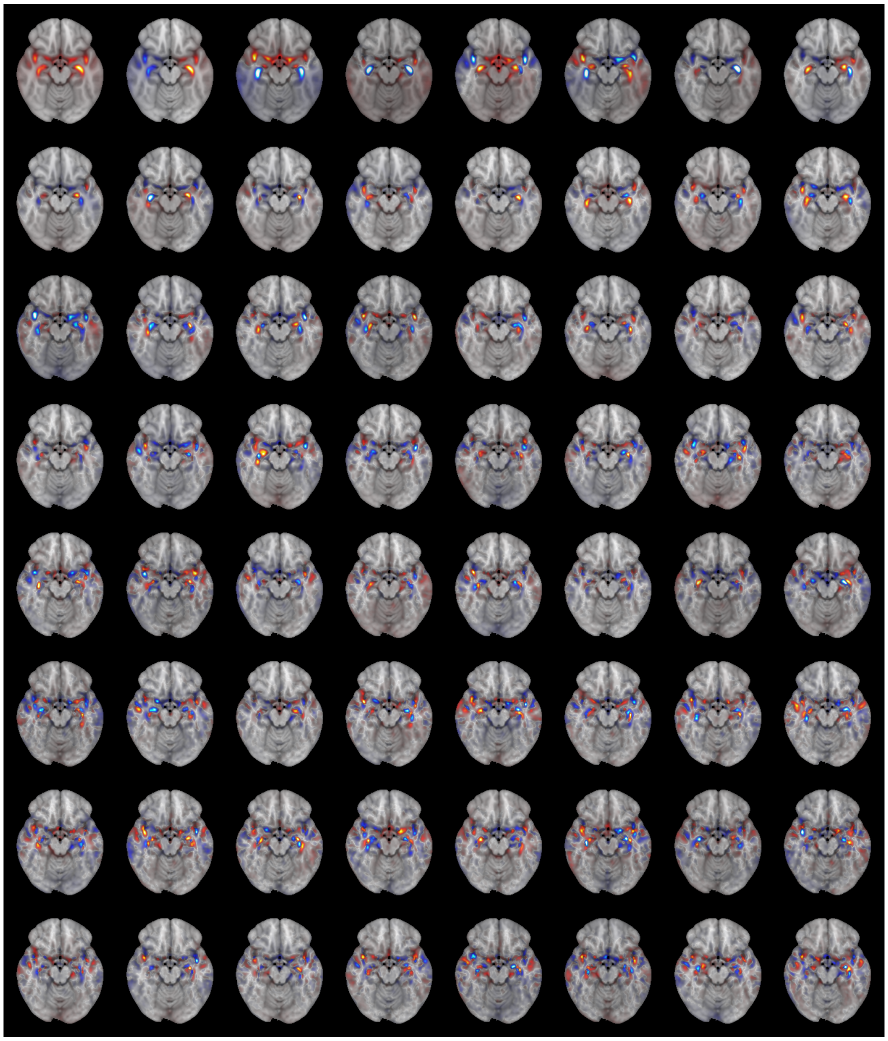

Supplementary Figure 6: A single axial slice from each of the 64 principal components plotted in brain space.

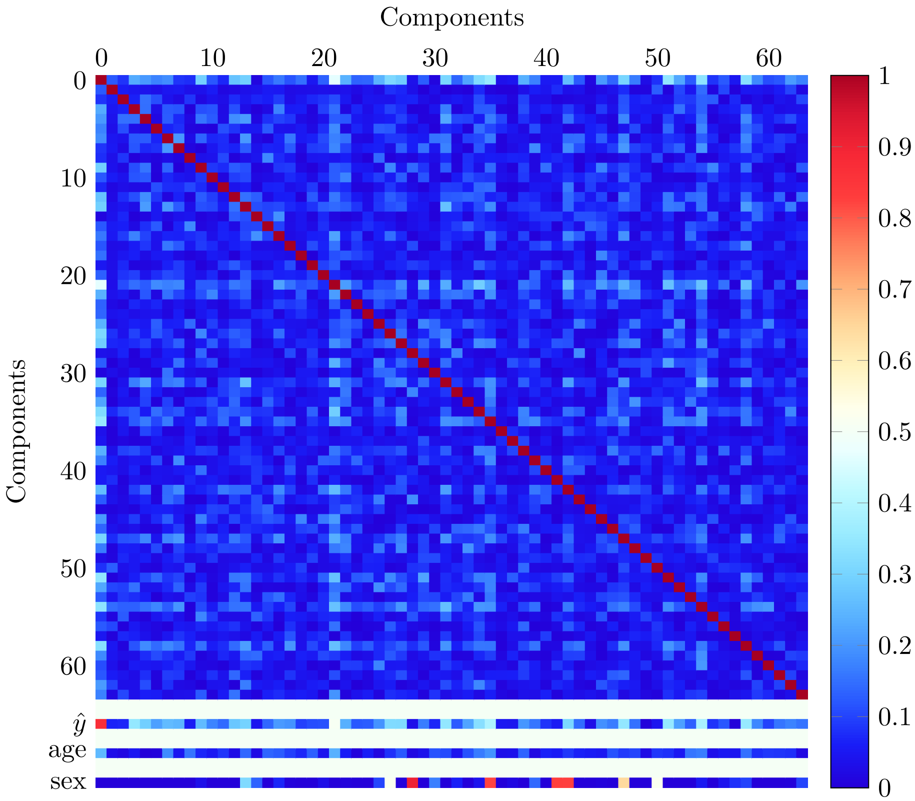

Supplementary Figure 7: Magnitudes of the correlations within the 64 principal components from the PCA, and between the components and prediction, age and sex. Correlations were computed in the dataset used for the exploratory MCI analyses.

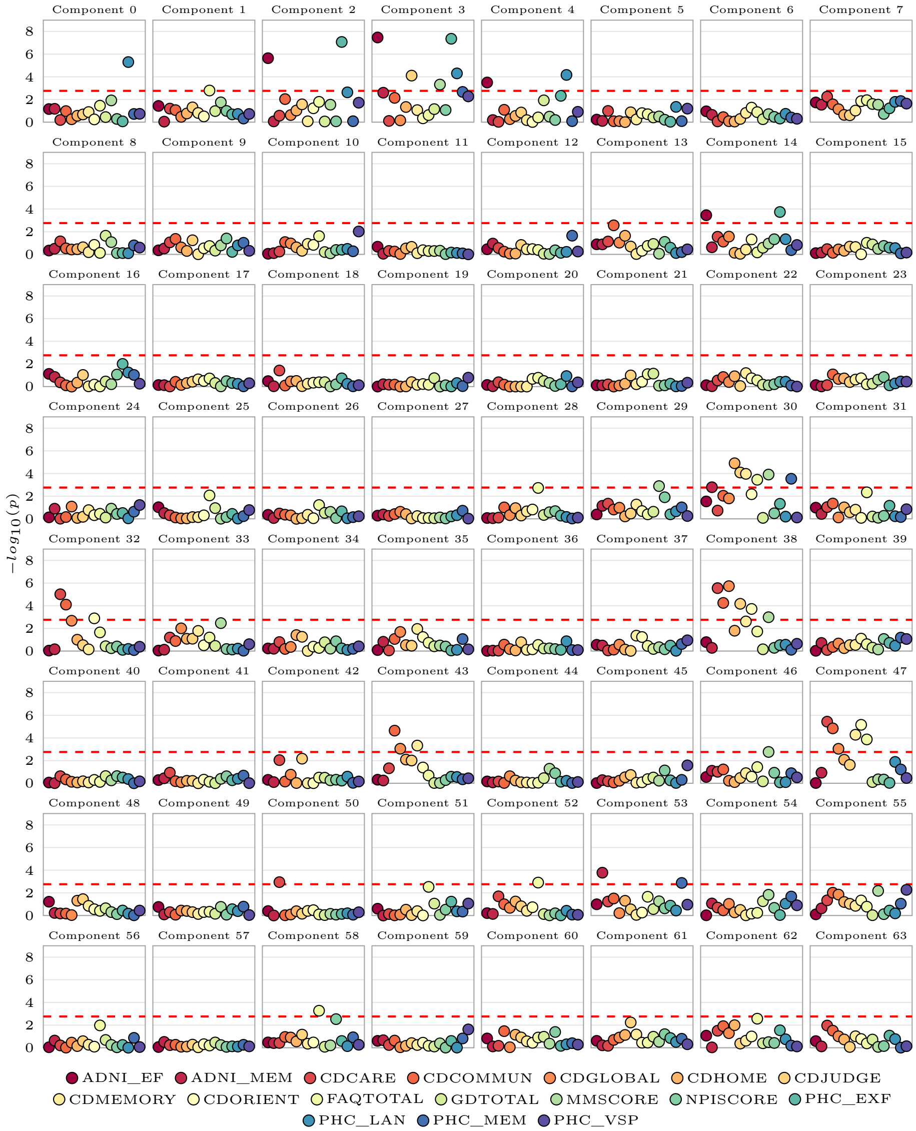

Supplementary Figure 8: P-values of the associations between the neuropsychological scores and each of the 64 components.

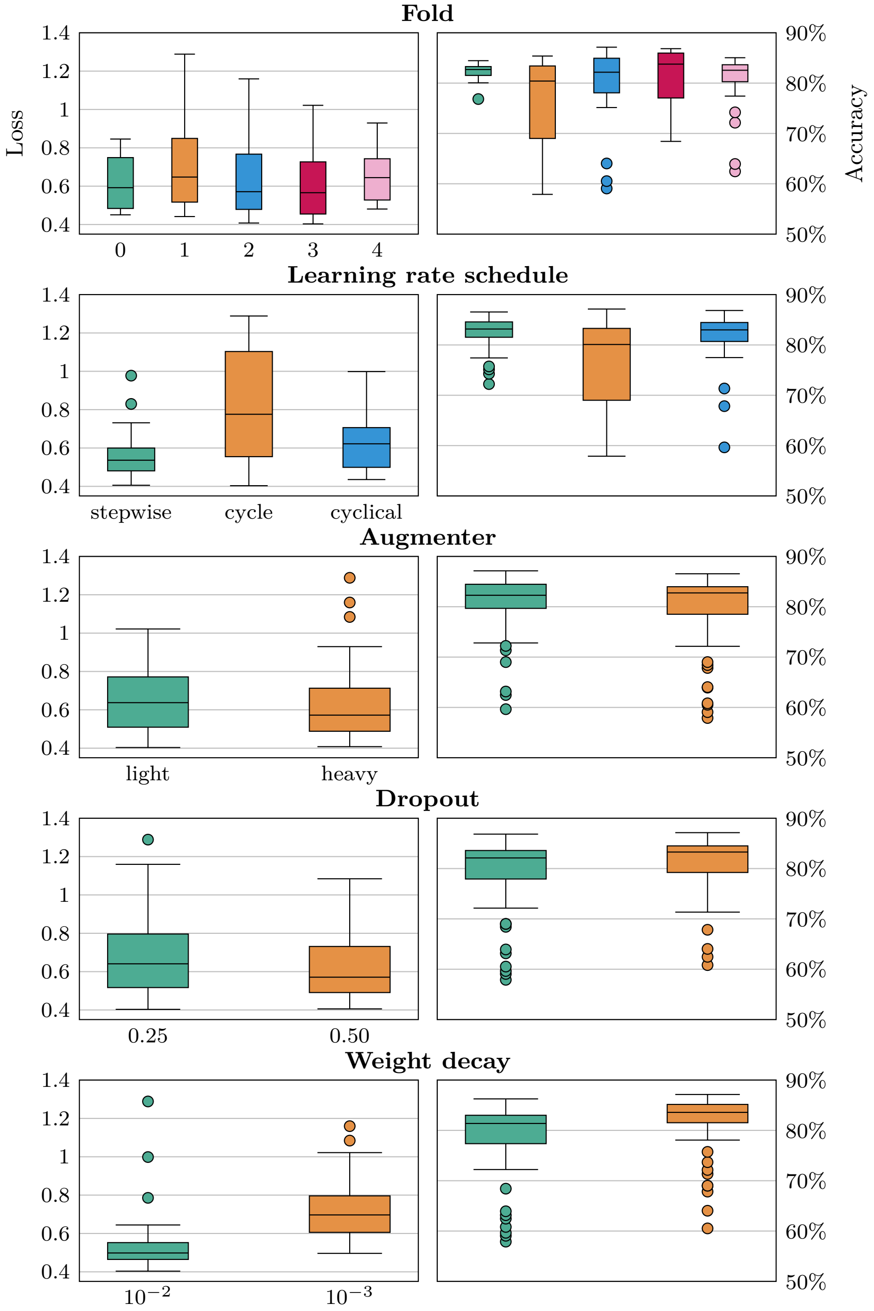

Supplementary Figure 9: Results achieved in the validation folds for the training runs of the dementia classifiers, grouped by each possible setting of each individual hyperparameter. For each box plot, the center line denotes the median, box edges the IQR, and whiskers the range of values, up to 1.5*IQR. Any points beyond this is shown as outliers.

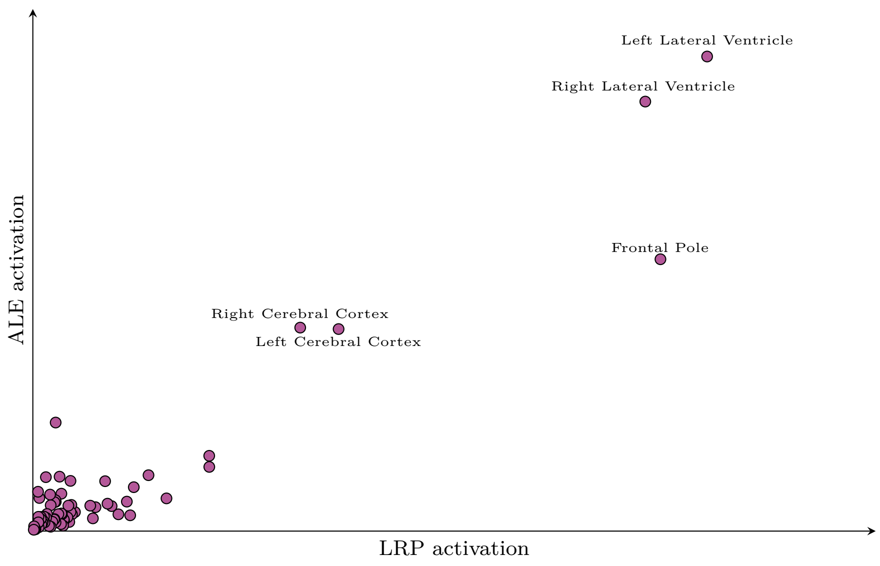

(a) Total activation per region

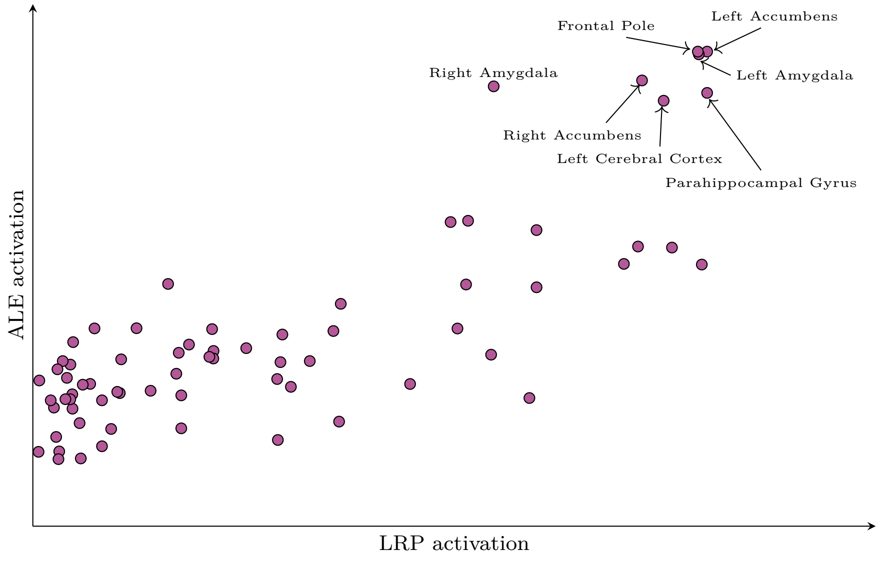

(b) Maximum activation per region

Supplementary Figure 10: Region-wise comparison of activation in the average map $\bar{R}_{dementia}$ produced by $LRP_{dementia}$ and G produced by the GingerALE meta-analysis, using different aggregation methods.

Cohort	Source	Comment	References
AddNeuroMed	Authors	AddNeuroMed consortium was led by Simon Lovestone, Bruno Vellas, Patrizia Mecocci, Magda Tsolaki, Iwona Kłoszewska, Hilkka Soininen. Their work was supported by InnoMed (Innovative Medicines in Europe), an integrated project funded by the European Union of the Sixth Framework program priority (FP6-2004- LIFESCIHEALTH-5)	[1, 2]
Alzheimer's Disease Neuroimaging Initiative	adni.loni.usc.edu	Data collection and sharing for this project was funded by the Alzheimer's Disease Neuroimaging Initiative (ADNI) (National Institutes of Health Grant U01 AG024904) and DOD ADNI (Department of Defense award number W81XWH-12-2-0012). ADNI is funded by the National Institute on Aging, the National Institute of Biomedical Imaging and Bioengineering, and through generous contributions from the following: AbbVie, Alzheimer's Association; Alzheimer's Drug Discovery Foundation; Araclon Biotech; BioClinica, Inc.; Biogen; Bristol-Myers Squibb Company; CereSpir, Inc.; Cogstate; Eisai Inc.; Elan Pharmaceuticals, Inc.; Eli Lilly and Company; EuroImmun; F. Hoffmann-La Roche Ltd and its affiliated company Genentech, Inc.; Fujirebio; GE Healthcare; IXICO Ltd.; Janssen Alzheimer Immunotherapy Research & Development, LLC.; Johnson & Johnson Pharmaceutical Research & Development LLC.; Lumosity; Lundbeck; Merck & Co., Inc.; Meso Scale Diagnostics, LLC.; NeuroRx Research; Neurotrack Technologies; Novartis Pharmaceuticals Corporation; Pfizer Inc.; Piramal Imaging; Servier; Takeda Pharmaceutical Company; and Transition Therapeutics. The Canadian Institutes of Health Research is providing funds to support ADNI clinical sites in Canada. Private sector contributions are facilitated by the Foundation for the National Institutes of Health (www.fnih.org). The grantee organization is the Northern California Institute for Research and Education, and the study is coordinated by the Alzheimer's Therapeutic Research Institute at the University of Southern California. ADNI data are disseminated by the Laboratory for Neuro Imaging at the University of Southern California.	[3, 4, 5]
Australian Imaging Biomarkers and Lifestyle flagship study of ageing	http://www.aibl.csiro.au/	Data used in the preparation of this article was obtained from the Australian Imaging Biomarkers and Lifestyle flagship study of ageing (AIBL) funded by the Commonwealth Scientific and Industrial Research Organisation (CSIRO) which was made available at the ADNI database (www.loni.usc.edu/ADNI). The AIBL researchers contributed data but did not participate in analysis or writing of this report. AIBL researchers are listed at www.aibl.csiro.au	[6]
Demgen	Authors	Supported by the Norwegian National Advisory Unit on Aging and Health	[7, 8]
MIRIAD	https://www.nitrc.org/projects/miriad/	Data used in the preparation of this article were obtained from the MIRIAD database (http://miriad.drc.ion.ucl.ac.uk). The MIRIAD investigators did not participate in analysis or writing of this report. The MIRIAD dataset is made available through the support of the UK Alzheimer's Society (Grant RF116). The original data collection was funded through an unrestricted educational grant from GlaxoSmithKline (Grant 6GKC).	[9]
OASIS3	https://www.oasis-brains.org/	Data were provided by OASIS-3: Longitudinal Multimodal Neuroimaging: Principal Investigators: T. Benzinger, D. Marcus, J. Morris; NIH P30 AG066444, P50 AG00561, P30 NS09857781, P01 AG026276, P01 AG003991, R01 AG043434, UL1 TR000448, R01 EB009352. AV-45 doses were provided by Avid Radiopharmaceuticals, a wholly owned subsidiary of Eli Lilly	[10]
StrokeMRI	Authors	Supported by the Research Council of Norway (249795, 248238), the South-Eastern Norway Regional Health Authority (2014097, 2015044, 2015073, 2016083), and the Norwegian ExtraFoundation for Health and Rehabilitation (2015/FO5146)	[11]
TOP	Authors	Supported by several grants from the Research Council of Norway, and the South-Eastern Norway Regional Health Authority	[12, 13, 14]

Supplementary Table 1: An overview of the datasets used in the present study.

**Dataset**	**Controls**	**Patients**
AddNeuroMed	MMSE $\geq$ 24	MMSE $<$ 19
ADNI	DX = CN	DX = AD
AIBL	DX = DXNORM	DX $\in$ {DXAD, DXOTHDEM}
Demgen	-	DX $\in$ {AD, OtherDem, UnspecDem, VaD}
MIRIAD	Group = Control	Group = AD
OASIS3	NORMCOG = 1	NORMCOG = 0 & DEMENTED = 1
StrokeMRI	Group = Control	-
TOP	diagnosis = CTRL	-

Supplementary Table 2: Criteria for inclusion in the case-control groups. For replicability, variable and category names are kept as they were originally used in the originating dataset.

	Validation		Test	
**Fold**	**AUC**	**Accuracy**	**AUC**	**Accuracy**
0	0.914	84.16%	0.917	83.04
1	0.929	84.79%	0.920	86.25
2	0.925	85.96%	0.915	87.13
3	0.929	87.42%	0.904	84.45
4	0.904	84.16%	0.905	83.87
	**0.918**	**85.30**	**0.908**	**84.95**

Supplementary Table 3: Predictive performance of the dementia classifiers split up into individual folds. The fold number denotes which fold was used as the test fold for the given run.

**Variable**	**Coefficient**	**p**
intercept	-0.373	1.94×10^{-3}
sex[M]	-0.084	7.29×10^{-4}
group[pMCI]	0.473	6.05×10^{-71}
age	0.009	8.76×10^{-10}
years_to_diagnosis	0.013	3.92×10^{-3}
years_to_diagnosis:group[pMCI]	0.050	8.14×10^{-17}

Supplementary Table 4: Coefficients and p-values from the linear mixed model modelling dementia prediction $\hat{y}$ as a function of various variables, including membership in the progressive (pMCI) or non-progressive group.

	Uncorrected		Corrected for $\hat{y}$	
**Component**	**p**	β	**p**	β
0	**1.60×10^{-66}**	0.683	**0.003**	0.18
1	0.061	−0.043	**0.001**	−0.07
2	**4.41×10^{-26}**	−0.248	**1.74×10^{-20}**	−0.22
3	**2.32×10^{-20}**	0.222	**9.49×10^{-10}**	0.16
4	**6.61×10^{-14}**	0.173	**3.91×10^{-09}**	0.14
5	0.117	−0.032	0.525	−0.01
6	**1.22×10^{-04}**	−0.083	**2.84×10^{-05}**	−0.09
7	0.11	−0.036	0.037	0.05
8	**2.90×10^{-04}**	0.076	**7.93×10^{-04}**	0.07
9	0.164	0.028	0.766	0.01
10	0.024	0.051	0.049	0.05
11	**5.74×10^{-05}**	0.082	**1.25×10^{-04}**	0.08
12	**3.50×10^{-05}**	−0.09	**2.78×10^{-06}**	−0.1
13	**0.003**	−0.063	**1.13×10^{-05}**	−0.1
14	**6.83×10^{-05}**	0.088	**0.003**	0.07
15	**1.94×10^{-04}**	−0.081	**1.91×10^{-06}**	−0.1
16	**0.001**	−0.071	0.016	−0.05
17	**6.44×10^{-05}**	−0.087	**0.001**	−0.07
18	**0.002**	−0.064	**9.70×10^{-07}**	−0.1
19	0.316	−0.022	0.152	−0.03
20	0.492	0.016	0.949	0.0
21	0.484	−0.018	0.374	−0.02
22	**0.008**	−0.067	**6.99×10^{-05}**	−0.1
23	**0.018**	0.049	0.068	0.04
24	**4.03×10^{-06}**	−0.106	**1.02×10^{-04}**	−0.09
25	**0.017**	0.057	**5.75×10^{-04}**	0.08
26	**1.40×10^{-07}**	−0.11	**3.63×10^{-07}**	−0.11
27	0.368	0.019	**0.006**	0.06
28	0.546	0.013	0.229	0.03
29	**4.03×10^{-04}**	−0.075	**2.80×10^{-04}**	−0.08
30	**3.44×10^{-08}**	−0.104	**1.72×10^{-15}**	−0.16
31	0.859	0.004	0.617	0.01
32	0.061	0.04	0.029	0.05
33	**0.003**	−0.063	**5.71×10^{-04}**	−0.08
34	0.059	0.04	**0.001**	0.07
35	0.316	−0.024	0.792	0.01
36	0.233	0.023	**0.007**	0.05
37	**1.82×10^{-05}**	−0.09	**5.41×10^{-06}**	−0.1
38	**7.93×10^{-04}**	0.075	**0.001**	0.07
39	0.267	−0.022	0.07	−0.04
40	**0.01**	0.052	**3.62×10^{-05}**	0.08
41	0.159	−0.034	0.08	−0.04
42	**1.87×10^{-07}**	−0.116	**2.83×10^{-10}**	−0.14
43	0.044	0.042	0.104	0.03
44	**0.009**	0.058	0.1	0.04
45	0.157	0.032	0.287	0.02
46	0.81	0.005	0.399	−0.02
47	**2.24×10^{-05}**	0.087	**0.001**	0.07
48	0.067	0.034	**0.009**	0.05
49	0.159	0.028	**0.009**	0.05
50	0.969	0.001	0.196	−0.02
51	**0.004**	0.065	**0.01**	0.06
52	0.691	0.008	0.424	0.02
53	0.805	−0.005	0.941	−0.0
54	0.204	0.032	0.071	0.05
55	0.082	0.033	0.121	0.03
56	0.207	0.028	0.104	0.04
57	0.147	−0.03	0.052	−0.04
58	**2.18×10^{-08}**	−0.11	**4.09×10^{-09}**	−0.12
59	0.05	0.035	**7.21×10^{-04}**	0.06
60	0.412	−0.017	0.173	−0.03
61	0.554	0.011	0.555	0.01
62	**8.24×10^{-04}**	0.067	0.023	0.05
63	0.336	0.019	**0.006**	0.05

Supplementary Table 5: Coefficient and p-value for each component in the two Partial Hazard analyses. In the corrected analyses (the two rightmost columns), the patients were stratified based on $\hat{y}$, to isolate the effect of the components.

Model	AUC	Balanced accuracy	PPV	Sensitivity	Specificity
$\mathcal{M}_{base}$	0.506±0.039	49.85%±3.7%	0.11±0.1	0.31±0.26	0.69±0.26
$\mathcal{M}_{pred}$	0.666±0.083	61.49%±10.58%	0.21±0.12	0.56±0.31	0.67±0.18
$\mathcal{M}_{comp}$	0.743±0.063	70.1%±4.79%	0.32±0.03	0.68±0.11	0.72±0.02

(a) One year

Model	AUC	Balanced accuracy	PPV	Sensitivity	Specificity
$\mathcal{M}_{base}$	0.474±0.05	49.47%±5.34%	0.33±0.05	0.47±0.06	0.52±0.07
$\mathcal{M}_{pred}$	0.742±0.053	69.92%±5.24%	0.52±0.07	0.75±0.09	0.65±0.08
$\mathcal{M}_{comp}$	0.786±0.019	74.31%±0.99%	0.61±0.04	0.72±0.04	0.76±0.06

(b) Two years

Model	AUC	Balanced accuracy	PPV	Sensitivity	Specificity
$\mathcal{M}_{base}$	0.536±0.032	53.9%±3.78%	0.32±0.26	0.35±0.29	0.73±0.23
$\mathcal{M}_{pred}$	0.797±0.037	75.76%±4.16%	0.75±0.04	0.76±0.09	0.76±0.06
$\mathcal{M}_{comp}$	0.808±0.016	77.17%±2.29%	0.79±0.04	0.72±0.03	0.82±0.05

(c) Three years

Model	AUC	Balanced accuracy	PPV	Sensitivity	Specificity
$\mathcal{M}_{base}$	0.529±0.056	53.01%±3.74%	0.38±0.32	0.35±0.29	0.71±0.24
$\mathcal{M}_{pred}$	0.844±0.041	80.6%±4.09%	0.84±0.04	0.82±0.06	0.79±0.04
$\mathcal{M}_{comp}$	0.867±0.031	80.38%±3.26%	0.87±0.02	0.75±0.08	0.85±0.02

(d) Four years

Model	AUC	Balanced accuracy	PPV	Sensitivity	Specificity
$\mathcal{M}_{base}$	0.515±0.031	51.05%±2.09%	0.14±0.28	0.09±0.18	0.93±0.14
$\mathcal{M}_{pred}$	0.889±0.024	83.61%±1.81%	0.91±0.02	0.83±0.04	0.84±0.03
$\mathcal{M}_{comp}$	0.903±0.034	84.1%±2.52%	0.92±0.02	0.82±0.03	0.86±0.04

(e) Five years

Supplementary Table 6: Predictive performance for each of the progression models at each of the five years following the MRI examination.

**Name**	**Included**	**Exclusion reason**
Alzheimer's Disease Assessment Scale Sub-scores	No	Missing ADNI2, 3, GO
ADSP Phenotype Harmonization Consortium	Yes	
Alzheimer's Disease Assessment Scale	No	Missing description
Clinical Dementia Rating	Yes	
Cognitive Change Index	No	Missing ADNI1, GO
Cogstate Battery	No	Missing ADNI1, GO
Cogstate Brief Battery	No	Missing ADNI1, 2, GO
Digital Cognitive Biomarkers	No	Missing summary scores
Everyday Cognition	No	Missing ADNI1
Financial Capacity Instrument	No	Missing ADNI1, 2, GO
Functional Activities Questionnaire	Yes	
Geriatric Depression Scale	Yes	
Item level data	No	Missing ADNI2, 3, GO
Mini-Mental State Examination	Yes	
Modified Haschinski Ischemia Scale	No	Non-congruent visit codes
Montreal Cognitive Assessment	No	Missing ADNI1
Neuropsychiatric Inventory Questionnaire	Yes	
Neuropsychological Battery	No	Missing explanation
UW - Neuropsych Summary Scores	Yes	

Supplementary Table 7: Neuropsychological assessments used as a basis for finding associations with the components from the relevance maps.

Variable	Source	Description
PHC_MEM	ADSP Phenotype Harmonization Consortium	Harmonized composite memory score
PHC_EXF	ADSP Phenotype Harmonization Consortium	Harmonized composite executive function score
PHC_LAN	ADSP Phenotype Harmonization Consortium	Harmonized composite language score
PHC_VSP	ADSP Phenotypxe Harmonization Consortium	Harmonized composite visuospatial score
CDRMEMORY	Clinical Dementia Rating	Clinical evaluation of impairment related to memory
CDRORIENT	Clinical Dementia Rating	Clinical evaluation of impairment related to orientation
CDRJUDGE	Clinical Dementia Rating	Clinical evaluation of impairment related to judgement and problem solving
CDRCOMMUN	Clinical Dementia Rating	Clinical evaluation of impairment related to community affairs
CDRHOME	Clinical Dementia Rating	Clinical evaluation of impairment related to home and hobbies
CDRCARE	Clinical Dementia Rating	Clinical evaluation of impairment related to personal care
CDRGLOBAL	Clinical Dementia Rating	Total clinical dementia rating
MMSCORE	Mini-Mental State Examination	Overall measure of cognitive impairment
FAQTOTAL	Functional Activities Questionnaire	Measures instrumental activities from daily life
GDTOTAL	Geriatric Depression Scale	Screening for depression in elderly adults
NPISCORE	Neuropsychiatric Inventory Questionnaire	Informant-based assessment of Neuropsychiatric symptoms
ADNI_EF	UW - Neuropsych Summary Scores	Composite score for executive functioning
ADNI_MEM	UW - Neuropsych Summary Scores	Composite score for memory

Supplementary Table 8: Test scores which were correlated against the components from the relevance maps.

**Hyperparameter**	**Values**
Dropout	{0.25, 0.5}
Weight decay	{1e-2, 1e-3}
Augmenter	{Light, Heavy}
Learning rate	{Stepwise, Cyclical, One-Cycle}

Supplementary Table 9: Hyperparameters that were tuned when training the dementia classifiers.

Supplementary references

[1] Yawu Liu et al. "Combination analysis of neuropsychological tests and structural MRI measures in differentiating AD, MCI and control groups–the AddNeuroMed study". In: *Neurobiology of Aging* 32.7 (July 2011), pp. 1198–1206. ISSN: 1558-1497. DOI: 10.1016/j.neurobiolaging.2009.07.008.

[2] S. Lovestone, P. Francis, and K. Strandgaard. "Biomarkers for disease modification trials–the innovative medicines initiative and AddNeuroMed". In: *The Journal of Nutrition, Health & Aging* 11.4 (2007), pp. 359–361. ISSN: 1279-7707.

[3] R. C. Petersen et al. "Alzheimer's Disease Neuroimaging Initiative (ADNI): clinical characterization". In: *Neurology* 74.3 (Jan. 19, 2010), pp. 201–209. ISSN: 1526-632X. DOI: 10.1212/WNL.0b013e3181cb3e25.

[4] Michael W. Weiner et al. "The Alzheimer's Disease Neuroimaging Initiative: a review of papers published since its inception". In: *Alzheimer's & Dementia: The Journal of the Alzheimer's Association* 8.1 (Feb. 2012), S1–68. ISSN: 1552-5279. DOI: 10.1016/j.jalz.2011.09.172.

[5] Michael W. Weiner et al. "The Alzheimer's Disease Neuroimaging Initiative 3: continued innovation for clinical trial improvement". In: *Alzheimer's & dementia : the journal of the Alzheimer's Association* 13.5 (May 2017), pp. 561–571. ISSN: 1552-5260. DOI: 10.1016/j.jalz.2016.10.006. URL: https://www.ncbi.nlm.nih.gov/pmc/articles/PMC5536850/ (visited on 06/01/2023).

[6] Kathryn A. Ellis et al. "The Australian Imaging, Biomarkers and Lifestyle (AIBL) study of aging: methodology and baseline characteristics of 1112 individuals recruited for a longitudinal study of Alzheimer's disease". In:

International Psychogeriatrics 21.4 (Aug. 2009), pp. 672–687. ISSN: 1041-6102. DOI: 10.1017/S1041610209009405.

[7] Aldo Córdova-Palomera et al. “Disrupted global metastability and static and dynamic brain connectivity across individuals in the Alzheimer’s disease continuum”. In: *Scientific Reports* 7.1 (Jan. 11, 2017), p. 40268. ISSN: 2045-2322. DOI: 10.1038/srep40268. URL: https://www.nature.com/articles/srep40268 (visited on 06/01/2023).

[8] Nhat Trung Doan et al. “Dissociable diffusion MRI patterns of white matter microstructure and connectivity in Alzheimer’s disease spectrum”. In: *Scientific Reports* 7 (Mar. 24, 2017), p. 45131. ISSN: 2045-2322. DOI: 10.1038/srep45131.

[9] Ian B. Malone et al. “MIRIAD–Public release of a multiple time point Alzheimer’s MR imaging dataset”. In: *NeuroImage* 70 (Apr. 15, 2013), pp. 33–36. ISSN: 1095-9572. DOI: 10.1016/j.neuroimage.2012.12.044.

[10] Pamela J. LaMontagne et al. *OASIS-3: Longitudinal Neuroimaging, Clinical, and Cognitive Dataset for Normal Aging and Alzheimer Disease*. Dec. 15, 2019. DOI: 10.1101/2019.12.13.19014902. URL: https://www.medrxiv.org/content/10.1101/2019.12.13.19014902v1 (visited on 06/01/2023).

[11] Erlend S. Dørum et al. “Age-related differences in brain network activation and co-activation during multiple object tracking”. In: *Brain and Behavior* 6.11 (Nov. 2016), e00533. ISSN: 2162-3279. DOI: 10.1002/brb3.533.

[12] Lars M. Rimol et al. “Cortical thickness and subcortical volumes in schizophrenia and bipolar disorder”. In: *Biological Psychiatry* 68.1 (July 1, 2010), pp. 41–50. ISSN: 1873-2402. DOI: 10.1016/j.biopsych.2010.03.036.

[13] Siren Tønnesen et al. "White matter aberrations and age-related trajectories in patients with schizophrenia and bipolar disorder revealed by diffusion tensor imaging". In: *Scientific Reports* 8.1 (Sept. 20, 2018), p. 14129. ISSN: 2045-2322. DOI: 10.1038/s41598-018-32355-9. URL: https://www.nature.com/articles/s41598-018-32355-9 (visited on 06/01/2023).

[14] Nhat Trung Doan et al. "Distinct multivariate brain morphological patterns and their added predictive value with cognitive and polygenic risk scores in mental disorders". In: *NeuroImage: Clinical* 15 (Jan. 1, 2017), pp. 719–731. ISSN: 2213-1582. DOI: 10.1016/j.nicl.2017.06.014. URL: https://www.sciencedirect.com/science/article/pii/S2213158217301456 (visited on 06/01/2023).
