# Supplementary material for: Constructing personalized characterizations of structural brain aberrations in patients with dementia using explainable artificial intelligence
Source: NPJ Digit Med. 2024 May 2;7:110. doi: 10.1038/s41746-024-01123-7 (PMC11066104; doi:10.1038/s41746-024-01123-7)
Supplement: Supplementary file 2 — Reporting Summary [file 41746_2024_1123_MOESM2_ESM.pdf]

nature portfolio

Corresponding author(s): Esten Høyland Leonardsen

Last updated by author(s): Feb 21, 2024

Reporting Summary

Nature Portfolio wishes to improve the reproducibility of the work that we publish. This form provides structure for consistency and transparency in reporting. For further information on Nature Portfolio policies, see our Editorial Policies and the Editorial Policy Checklist.

Statistics

For all statistical analyses, confirm that the following items are present in the figure legend, table legend, main text, or Methods section.

n/a	Confirmed	
☐	☒	The exact sample size (*n*) for each experimental group/condition, given as a discrete number and unit of measurement
☐	☒	A statement on whether measurements were taken from distinct samples or whether the same sample was measured repeatedly
☐	☒	The statistical test(s) used AND whether they are one- or two-sided<br>*Only common tests should be described solely by name; describe more complex techniques in the Methods section.*
☐	☒	A description of all covariates tested
☐	☒	A description of any assumptions or corrections, such as tests of normality and adjustment for multiple comparisons
☒	☐	A full description of the statistical parameters including central tendency (e.g. means) or other basic estimates (e.g. regression coefficient) AND variation (e.g. standard deviation) or associated estimates of uncertainty (e.g. confidence intervals)
☒	☐	For null hypothesis testing, the test statistic (e.g. *F*, *t*, *r*) with confidence intervals, effect sizes, degrees of freedom and *P* value noted<br>*Give P values as exact values whenever suitable.*
☒	☐	For Bayesian analysis, information on the choice of priors and Markov chain Monte Carlo settings
☐	☒	For hierarchical and complex designs, identification of the appropriate level for tests and full reporting of outcomes
☐	☒	Estimates of effect sizes (e.g. Cohen's *d*, Pearson's *r*), indicating how they were calculated

Our web collection on statistics for biologists contains articles on many of the points above.

Software and code

Policy information about availability of computer code

Data collection	The data used in this study were gathered from various sources, an overview including acknowledgements of their respective funding sources is provided in the Supplementary Materials.
Data analysis	FreeSurfer 5.3, FSL 6.0, Python 3.9, Tensorflow 2.6, scikit-learn 1.2, statsmodels 0.13. In addition public repositories developed by the authors available at https://github.com/estenhl/pyment-public and https://github.com/estenhl/keras-explainability. The latter two contains code for deep learning modelling and explainability

For manuscripts utilizing custom algorithms or software that are central to the research but not yet described in published literature, software must be made available to editors and reviewers. We strongly encourage code deposition in a community repository (e.g. GitHub). See the Nature Portfolio guidelines for submitting code & software for further information.

Data

Policy information about availability of data

All manuscripts must include a data availability statement. This statement should provide the following information, where applicable:

- Accession codes, unique identifiers, or web links for publicly available datasets
- A description of any restrictions on data availability
- For clinical datasets or third party data, please ensure that the statement adheres to our policy

The data used in this study was obtained from the Alzheimer's Disease Neuroimaging Initiative (adni.loni.usc.edu for further details), the Australian Imaging

Biomarkers and Lifestyle flagship study of ageing (www.aibl.csiro.au), MIRIAD (www.nitrc.org/projects/miriad), and OASIS (https://www.oasis-brains.org/). In addition to these we used data from the AddNeuroMed consortium, Demgen, TOP and StrokeMRI, previously published studies that can be accessed via the principal investigators.

Research involving human participants, their data, or biological material

Policy information about studies with human participants or human data. See also policy information about sex, gender (identity/presentation), and sexual orientation and race, ethnicity and racism.

Reporting on sex and gender	Results apply to both sexes. The predictive deep learning models were trained and tested on participants of both sexes. In subsequent analyses sex was included as a covariate for correction.
Reporting on race, ethnicity, or other socially relevant groupings	No socially constructed or socially relevant variables were explicitly included in the analyses.
Population characteristics	The included were participants with ages ranging from 54 to 97 years that were either diagnosed with dementia or mild cognitive impairment, or collected as healthy controls with matching population characteristics
Recruitment	The data used in the present study originates from multiple data collection efforts and studies with varying recruitment strategies.
Ethics oversight	The use of Demgen, TOP and StrokeMRI data have been approved via Regional Committes for medical and health research ethics (REK) in Norway. The use of the remaining datasets happen in agreement with their data usage agreements, and for AddNeuroMed, ADNI and AIBL after explicit approval from their principal investigators.

Note that full information on the approval of the study protocol must also be provided in the manuscript.

Field-specific reporting

Please select the one below that is the best fit for your research. If you are not sure, read the appropriate sections before making your selection.

☐ Life sciences ☒ Behavioural & social sciences ☐ Ecological, evolutionary & environmental sciences

For a reference copy of the document with all sections, see nature.com/documents/nr-reporting-summary-flat.pdf

Behavioural & social sciences study design

All studies must disclose on these points even when the disclosure is negative.

Study description	A quantitative study based on predictive modelling using deep neural networks
Research sample	A combined dataset compiled from multiple previously published studies. There was not collected any new data for this study
Sampling strategy	n/a
Data collection	n/a
Timing	n/a
Data exclusions	n/a
Non-participation	n/a
Randomization	n/a

Reporting for specific materials, systems and methods

We require information from authors about some types of materials, experimental systems and methods used in many studies. Here, indicate whether each material, system or method listed is relevant to your study. If you are not sure if a list item applies to your research, read the appropriate section before selecting a response.

Materials & experimental systems

n/a	Involved in the study	
☒	☐	Antibodies
☒	☐	Eukaryotic cell lines
☒	☐	Palaeontology and archaeology
☒	☐	Animals and other organisms
☒	☐	Clinical data
☒	☐	Dual use research of concern
☒	☐	Plants

Methods

n/a	Involved in the study	
☒	☐	ChIP-seq
☒	☐	Flow cytometry
☐	☒	MRI-based neuroimaging

Plants

Seed stocks: n/a

Novel plant genotypes: n/a

Authentication: n/a

Magnetic resonance imaging

Experimental design

Design type: Structural MRI

Design specifications: n/a

Behavioral performance measures: n/a

Acquisition

Imaging type(s): Structural

Field strength: 1.5T and 3T

Sequence & imaging parameters: Multiple acquisition protocols

Area of acquisition: Whole brain scans

Diffusion MRI: ☐ Used ☒ Not used

Preprocessing

Preprocessing software: FreeSurfer 5.3, FSL 6.0

Normalization: Linear transformation with six degrees of freedom using FSL flirt

Normalization template: MNI152

Noise and artifact removal: None

Volume censoring: None

Statistical modeling & inference

Model type and settings: Deep neural networks

Effect(s) tested: n/a

Specify type of analysis: ☒ Whole brain ☐ ROI-based ☐ Both

Statistic type for inference (See Eklund et al. 2016)	Voxel-wise analysis of minimally preprocessed whole brian scans
Correction	For a subset of the analyses were multiple tests were performed, FDR correction was used

Models & analysis

n/a	Involved in the study
☒	☐ Functional and/or effective connectivity
☒	☐ Graph analysis
☐	☒ Multivariate modeling or predictive analysis

Multivariate modeling and predictive analysis	The raw imaging data was used to train deep neural networks
